# Profile: Professor Philippa Garety

**DOI:** 10.1192/pb.bp.115.051367

**Published:** 2016-04

**Authors:** Julia Bland

## Abstract

Healthy professional one-upmanship is exemplified in Philippa Garety's position as a professor of clinical psychology, a clinical director and a joint leader of a psychosis clinical academic group. Julia Bland sought to discover whether psychiatrists have anything substantial to offer that psychologists cannot.

**Figure:**
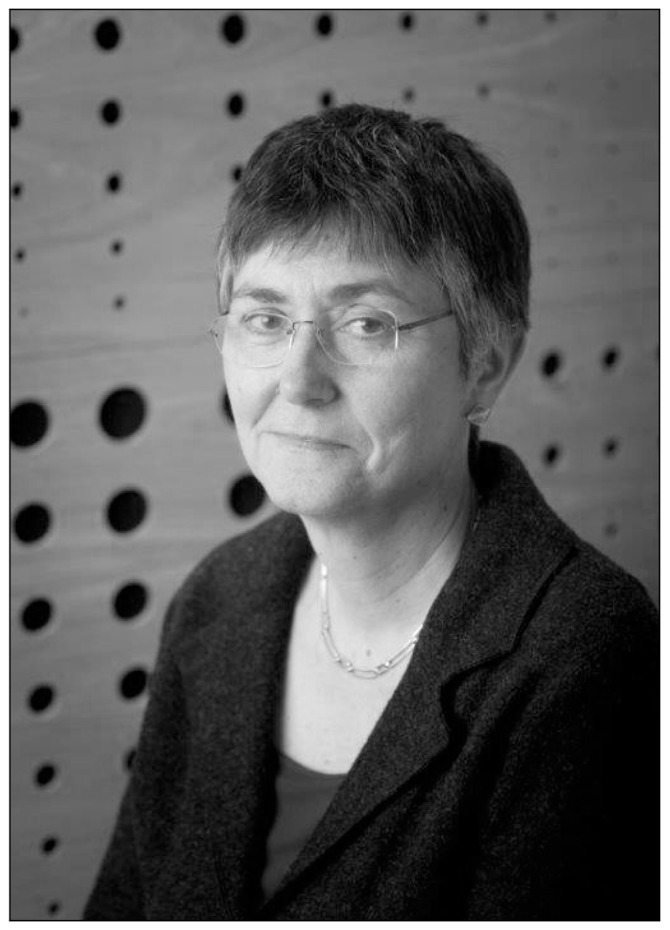
Professor Philippa Garety, Clinical Director and Joint Leader, Psychosis Clinical Academic Group

Philippa Garety sounds like she ought to be quite a cosy, girlish, slightly old fashioned, innocent granny in a rocking chair. You couldn't be more wrong. She is a ground breaker, albeit in a polite, non-ball breaker way. She is not really interested in self-promotion. And was slightly anxious, and wanting to check my copy before it went out. She does not want to make waves, certainly not destructive ones, and is deeply serious about her role and responsibility, without any grandiosity.

Her preferred modus operandi is harmonious: she leads, jointly, the biggest clinical academic group in one of the biggest mental health trusts in the UK (South London and Maudsley NHS Foundation Trust; there are approximately 5000 patients under the care of recovery teams, about 1000 under early intervention, and another 1000 in complex care/rehabilitation, Trust-wide). Her style rests on negotiation, mutual respect, scientific evidence, consensus. But make no mistake, she is not a pushover and has the ideas, vision and the ‘intellectual self-confidence’ (as she put it) to have taken on this new and high-profile role.

Instinctively collaborative, she was attracted to the job by the triumvirate power-sharing. She is the clinical lead, with an academic lead, Professor Philip McGuire, and a service lead, Lucy Canning, and they work closely together. The shared leadership model attracted her as a way of getting away from a medical director with clinical responsibility often ‘keeping distance from the service director in order to be able to critique the service director’. She genuinely believes that the new collaborative style of leadership is ‘more supportive of innovative and excellent clinical practice with a strategic focus’. They are trying to improve delivery of mental health services across the psychosis spectrum, from early onset, first episode, through longer term and recovery. The approach is non-doctrinaire, pragmatic, aiming for excellence in a cash-strapped health environment, not a task for the faint hearted. It has meant convincing staff, managers and finally commissioners that continual cutting of funding has the perverse consequence of increasing costs, by increasing instability and bed usage, and overwhelming staff who in turn become less effective. They have pulled off a piece of magic, successfully persuading local commissioners that investment is more effective at cost containment than disinvestment: boosting community services, along the lines successful with early intervention for psychosis, with a view to reducing bed usage and thus costs in the longer term – ‘It's early days, but bed usage is going in the right direction’. Strategy needs to be logical: ‘I know data can be used in different ways, and that there is no simple identity between data and reality, but over the years we have seen decisions being made which are highly politicised and/or pragmatic, without really looking for the information.’

There is ‘some evidence’ for early intervention ‘when case-loads are sensible, there is good leadership, the best range of interventions is available and staff are sufficiently skilled, and supervised to offer them’. She also emphasised the need for ‘understanding about the importance of engagement, working with families and carers, excellent medical intervention, vocational specialists’. And how often is that the case on the ground, I wondered?

We talked about the evidence for supportive generic outreach versus more specific and formal structured interventions. She acknowledged the importance of continuity in the relationship between service user and service deliverer, whatever they think they are delivering. She also agreed that the sophisticated nurse, psychologist or psychiatrist actually uses a range of therapeutic skills within any one consultation, which are difficult to measure.

Nevertheless, it is clear that she respects scientific research methodology: it may be difficult to ask the right questions, and to deliver robust answers to questions about what is most useful and why, but this does not obviate the need to try to get the most reliable answers we can, and base policy on those rather than on mere hunch.

Over the course of our conversation, I came to the conclusion that her drivers are simple and unfashionable: compassion and a passion to provide the optimum treatment (psychological, social or pharmacological) for people with psychosis, who, as we all know, suffer enough without incompetent service delivery. Philippa Garety is not preachy, and she certainly did not directly state this motivation. But it shone through her lack of vainglory.

She was brought up in Mill Hill, in North London, and was sent to a convent by her Catholic parents. Her Christian roots have moved to the Church of England, and although it felt too intrusive to press her on theological matters, I got a sense that this is solid and important to her. At Cambridge, she studied philosophy for 2 years, with the intention of becoming a lawyer, but decided it would be ‘too boring’. She came to the (then) Institute of Psychiatry to become a clinical psychologist in 1979, and perhaps it is not entirely coincidental that this institution has recently been renamed the full mouthful of the Institute of Psychiatry, Psychology and Neuroscience.

I wanted to know how philosophy had informed her thinking as a psychologist. She has described her curiosity being piqued, when, as a young psychologist, a patient stated baldly, ‘You put my mother in the washing machine’.

She brings epistemological questions to bear on psychotic experience – ‘how can we claim to know what we know?’ Wittgenstein and linguistic philosophy was the hot area during her time in Cambridge, but she was drawn to the philosophy of science and epistemology; how different models of understanding arise, how meaning is attributed. Personally, she is a flexible thinker, able to see different perspectives, and is allergic to the doctrinaire: ‘one can operate within certain frameworks, as long as they seem useful, but they don't have to be seen as representing single notions of reality’. Perhaps this flexibility has enhanced her interprofessional capacities? Working, as she does, constantly, with psychiatrists, described as ‘my closest colleagues’.

## Interprofessional rivalry: why bother with psychiatrists?

Philippa Garety's sweetly reasonable stance did not make for an exciting discussion of interprofessional rivalry. Being interviewed for a psychiatric journal aside, I failed to elicit any personal animus towards psychiatrists.

She points out that historically clinical leadership has been attached to psychiatry and nursing rather than psychology, and ‘there are far more of you’. She almost makes me feel sorry for psychologists who she claims are ‘more inclined to lead other psychologists, and psychological services, but not so comfortable with other professionals’. I wonder whether she could be referring to us overconfident medics.

We discussed the unequivocal challenge to psychiatric hegemony from Allan S. Mariner, an American psychologist, who sees medical training as largely irrelevant to mental health practice:

‘in the mental health field … only three professional activities … are firmly operationally connected with medical training: performing physical examinations, prescribing drugs, and giving electroshock treatment … the mental health practitioner, whether he be psychiatrist, psychologist, psychiatric social worker, or lay analyst, is *basically practising applied psychology* [my italics]. A truly relevant curriculum leading to a doctorate in mental health must be developed from the curriculum in clinical psychology, not the medical curriculum’.^1^

Neuropsychiatry and liaison psychiatry aside, does Dr Mariner have a point? But Professor Garety is too tactful to make any superior claims for psychology (in spite of the better research training we do agree that psychology trainees receive).

In her peacable world, promotion should be based simply on the best person for the job, profession immaterial. Although this is eminently sensible, it does not describe the reality of mutual suspicion, ignorance, rivalry and disrespect, which I have seen in several dark corners of mental health provision.

Her one criticism of contemporary clinical psychology was the lack of social mix in the intake to training. It is so hard to be accepted for clinical psychology training that the system inevitably favours those whose parents can support them while they do unpaid work to spruce up their CVs. ‘It's the internship argument’, she says. I suggest that the preponderance of middle-class blonde women becoming clinical psychologists does not make for a profession that mirrors the diversity of the service users they serve, and she agrees that there is a worrying bias in gender and class.

## CBT for psychosis

But Professor Garety has not entirely avoided controversy. As a leading researcher in the psychological treatment of psychosis, she was instrumental in the National Institute for Health and Care Excellence guidelines recommending cognitive-behavioural therapy (CBT) and family intervention for psychosis (2003, updated 2014).^2^ Later studies have attacked the effectiveness of CBT for psychosis in preventing relapse. She concedes this may be true, but still supports the symptomatic relief that CBT affords. ‘It is fairly clear on about 12 meta-analyses that CBT does offer benefit on a range of outcomes with a small- to medium-effect size … Oddly enough it seems that CBT is better for symptom relief but not so effective in preventing relapse, while [family intervention] is the other way round.’

So is it still worth trying to roll out CBT? An unequivocal yes: ‘But of course it's not a panacea. The next generation of CBT will be more targeted for the individual, addressing command hallucinations, paranoia or depression more specifically, I hope’. She also sees future trials as being more targeted to specific symptoms.

One of her own main research interests is the emotional and cognitive context of delusions, clearly still fuelled by being intrigued by the philosophical as well as clinical questions around derivation of meaning for the individual. She still sees patients one afternoon a week.

Her empathic and sensible view is of circular reinforcement: anxiety increases the negative appraisal of self and others, promoting paranoid thoughts, which in turn increase anxiety. Social isolation and adverse environments clearly play into this cycle.

The clinician's attitude to the person with psychosis should be validating and normalising: the ‘anyone who had been through what you've been through’ approach, relating to the patient as someone who has experienced adversity and is coping with confusing and distressing experiences. The mental health professional has to be not only non-colluding but also non-confrontative, attempting to develop a collaborative understanding with the patient of the patient's own thinking. Easier said than done.

Her service-based research includes a randomised controlled trial of community-based assertive outreach in first-episode psychosis, with Professor Tom Craig. She has looked at the psychological effect of the deprived urban environment on individuals with persecutory delusions, and at how factors such as increased social support in a low expressed emotion environment can increase the flexibility of delusional belief.

What about life beyond psychology and psychosis? Her happiest weekends are full of family and fresh air: she and her partner (a woman and Anglican priest) sail, walk and spend time with the extended family. Philippa is the youngest of six, several of whom are also keen sailors and have houses in the same village on the Hampshire coast. Generally upbeat, she minimises the difficulties she has experienced around coming out as gay in a Catholic family, but admits that it has got easier as she has got older.

Family life is not all play: the long haul of helping her elderly parents maintain as much independence for as long as possible has taken its toll.

Her unlived life is that of a farmer, but in her world after psychology, she will settle for an orchard, although ‘definitely not fiddling with making jam’. From the perspective of her current busy professional life, she does harbour a pull towards a more contemplative, outdoors life, with more room for silence: ‘I think I'm quite an introvert’. All the more decent then, of this thoughtful, intelligent and energetic woman, to place her time at the disposal of a hectic organisation which demands constant interaction with people, psychotic and otherwise.
